# The Prognosis of Leptin rs2167270 G > A (G19A) Polymorphism in the Risk of Cancer: A Meta-Analysis

**DOI:** 10.3389/fonc.2021.754162

**Published:** 2021-11-18

**Authors:** Aiqiao Zhang, Shangren Wang, Fujun Zhang, Wei Li, Qian Li, Xiaoqiang Liu

**Affiliations:** ^1^ Department of Neonatology, First Teaching Hospital of Tianjin University of Traditional Chinese Medicine, Tianjin, China; ^2^ Department of Neonatology, National Clinical Research Center for Chinese Medicine Acupuncture and Moxibustion, Tianjin, China; ^3^ Department of Urology, Tianjin Medical University General Hospital, Tianjin, China

**Keywords:** leptin (LEP), cancer, polymorphism, A19G, rs2167270

## Abstract

**Background:**

Although the effect of the LEP G19A (rs2167270) polymorphism on cancers is assumed, the results of its influence have been contradictory. A meta-analysis was conducted to precisely verify the relationships between LEP G19A and the development of digestion-related cancers.

**Methods:**

Investigators systematically searched the literature in PubMed, Embase, and Web of Science and used STATA software 14.0 for the meta−analysis. The odds ratios (ORs) and 95% confidence intervals (CIs) were calculated to evaluate the associations. Subgroup analyses stratified by ethnicity, cancer type, and cancer system were further conducted to assess the relationship between the LEP G19A polymorphism and digestion-related cancers.

**Results:**

In the overall population, we found a significant relationship with overall cancer (allele comparison: OR = 0.921, *p* = 0.000; dominant comparison: OR = 0.923, *p* = 0.004; recessive comparison: OR = 0.842, *p* = 0.000; homozygote model: OR = 0.0843, *p* = 0.001). In a subgroup analysis conducted by ethnicity, we obtained significant results in Asians (Asian allele comparison: OR = 0.885, *p* = 0.000; dominant comparison: OR = 0.862, *p* = 0.000; homozygote model: OR = 0.824, *p* = 0.039; and heterozygote comparison: OR = 0.868, *p* = 0.000) but not in Caucasians. In a subgroup analysis conducted by cancer type and cancer system, we obtained significant results that the LEP G19A polymorphism may decrease the risk of colorectal cancer, esophageal cancer, digestive system cancer, and urinary system cancer.

**Conclusions:**

This meta-analysis revealed that the LEP G19A polymorphism may decrease the risk of cancer.

## Introduction

It is well known that cancer is one of major causes of death with over 6.1 million projected to die each year, and morbidity rates have increased gradually over the past decade (1, 2), so it has been a public health burden worldwide. The reason for cancer is complicated and the etiology and mechanism of carcinogenesis are not clearly elucidated to date. It was widely accepted that the interplay between environmental factors, genetics, and lifestyle plays an important role in the carcinogenesis according to epidemiology. There is mounting evidence indicating that many metabolic diseases such as obesity and diabetes may significantly increase the risk of cancer (3–5). The polymorphism of obesity and diabetes gene may be associated with genetic susceptibility of cancer.

Leptin (LEP), a 16-kDa hormone of energy expenditure, is a balancing mediator of homeostasis by regulating acquisition and consumption of energy, which was a basic pathophysiological process in normal cells and cancer cells. Many epidemiological studies have revealed the link between LEP and the development of many kinds of cancers (6–8). Among the pathophysiological mechanisms of cancer, LEP seems relevant to the proliferation of cancer stem cells (9). Some studies also revealed that LEP through its signal pathways regulating energy intake and expenditure [MAPK, PI3K, mTOR, and JAK/STAT (10, 11)] produced an effect in angiogenesis processes that were critical in the genesis and development of cancer (12). Pathophysiological mechanisms of cancer such as inflammation, invasion, and metastasis are also favored by LEP (13–15). So, LEP may be involved in various pathological processes of carcinogenesis.

Single-nucleotide polymorphism can change the functions of genes and the expression of protein. LEP G19A polymorphism, positioning at the 5′-untranslated region of gene, may impact mRNA translation and change the serum level of LEP. With the development of molecular epidemiology, various studies have demonstrated that LEP G19A polymorphism is related to cancer risk (16–19). However, results between G19A polymorphism with cancers have been inconsistent or inconclusive. Therefore, we performed a meta­analysis to verify the correlation between the G19A mutation of the LEP gene and susceptibility to cancers.

In this study, we conducted a meta-analysis to verify whether the G19A polymorphism of the LEP gene affects the risk of cancer.

## Methods

### Literature Search

A comprehensive literature search of PubMed, Embase, and Web of Science was performed to search all potential studies that involved the relevance between the G19A polymorphism and cancers prior to June 2021. Our study contained the following terms: (“leptin” OR “LEP” OR “G19A” OR “rs2167270”) AND (“polymorphism” OR “variant” OR “mutation”) AND (“malignancy” OR “cancer” OR “carcinoma” OR “neoplasm”).

### Inclusion and Exclusion Criteria

The inclusion criteria were as follows: (1) investigate the association between the LEP G19A (rs2167270) mutation and cancers; (2) meet cohort design or case–control design; (3) abundant data should behave to estimate an odds ratio (OR) and 95% confidence interval; (4) results were reported in English; and (5) include human subjects. We adopted the following exclusion criteria: (1) duplicated studies; (2) studies in which subjects were not human; and (3) studies in which we could not obtain sufficient raw data.

### Data Extraction

Investigators extracted genotype data independently, and every data point reached a consensus. The extracted data contained the (1) name of the first author; (2) year of publication; (3) ethnicity of cases and controls; (4) cancer type of studies; and (5) frequency of LEP G19A in genes.

### Statistical Analysis

We computed ORs and their 95% CIs to estimate the association between the LEP G19A (rs2167270) mutation and cancers. The pooled ORs and their 95% CIs were computed for genes using the following five models: dominant model (AA + AG vs. GG), recessive model (AA vs. GG + AG), allele model (A vs. G), homozygous model (AA vs. GG), and heterozygote model (AG vs. GG).

The *Q* test was used to estimate heterogeneity between different studies, and *p* < 0.05 was considered significant for heterogeneity. In addition, inconsistency was quantified by the *I* ^2^ statistic. Twenty-five percent and 50% of the *I* ^2^ values indicated low and high levels of heterogeneity, respectively. An *I* ^2^ < 50% suggested that no heterogeneity existed. When heterogeneity existed, the fixed effects model (FEM) was utilized; otherwise, the random-effects model (REM) was utilized for calculation.

To evaluate the specific effects of ethnicity, cancer type, and cancer system, investigators performed subgroup analyses by ethnicity, cancer type, and cancer system.

Sensitivity analyses were performed to evaluate the stability of the results. A funnel plot of Egger’s or Begg’s test was conducted to reveal possible publication bias. We used the Newcastle-Ottawa Scale to assess the including literature quality. All meta-analyses were performed using STATA software (Version 12.0, College Station, TX).

## Results

### Study Characteristics

**Figure 1:**
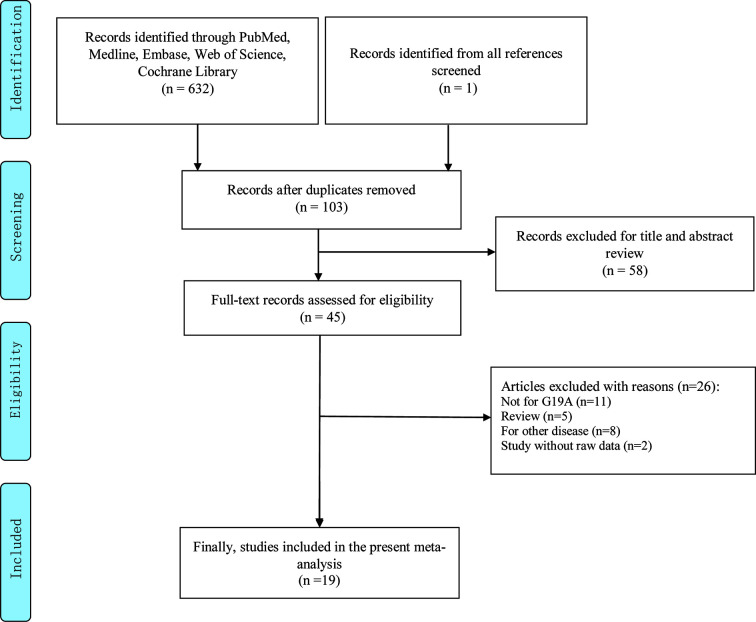
Flowchart of literature selection.

Depending on the search strategy, 633 articles were retrieved (**Figure 1**). Among them, 103 articles were eligible after excluding repeated publications. By reviewing the titles and study abstracts, 58 articles were excluded. Of the remaining 45 studies, 26 articles were excluded, including 11 studies that were not focused on the LEP G19A genetic mutation. Five studies were meta-analyses. Eight studies were on other disorders that were not cancer. Two articles did not provide raw data. Finally, 19 studies conformed to our meta-analyses, and **Tables 1** and **2** summarize the extracted data (16–34).

**Table 1:** The characters of included studies in the meta-analysis.

Author	Year	Country	Ethnicity	Cancer type	Cancer system	Genotype	
						Case	Control
Skibola et al. (16)	2004	USA	Caucasians	Non-Hodgkin lymphoma	Hematopoietic malignancy	373	805
Willett et al. (17)	2005	UK	Caucasians	Non-Hodgkin lymphoma	Hematopoietic malignancy	590	754
Slattery et al. (18)	2008	USA	Mixed	Colorectal cancer	Digestive system cancer	1,567	1,965
Doecke et al. (20)	2008	Australia	Caucasians	Esophageal cancer	Digestive system cancer	774	1,352
Tsilidis et al. (19)	2009	USA	Mixed	Colorectal cancer	Digestive system cancer	204	362
Wang et al. (21)	2009	USA	Caucasians	Prostate cancer	Urinary system cancer	253	257
Moore et al. (22)	2009	Finland	Caucasians	Prostate cancer	Urinary system cancer	945	840
Partida-Perez et al. (23)	2010	Mexico	Caucasians	Colorectal cancer	Digestive system cancer	68	102
Kim et al. (24)	2012	Korea	Asian	Breast cancer	Others	400	452
Zhang et al. (25)	2012	China	Asian	Non-Hodgkin lymphoma	Hematopoietic malignancy	514	557
Qiu et al. (26)	2017	China	Asian	Esophageal cancer	Digestive system cancer	502	1,496
Zhang et al. (27)	2018	China	Asian	Hepatocellular carcinoma	Digestive system cancer	584	923
Huang et al. (28)	2018	USA	Mixed	Colorectal cancer	Digestive system cancer	134	259
Yang et al. (29)	2019	China	Asian	Esophageal cancer	Digestive system cancer	1,063	1,677
Lin et al. (30)	2020	China	Asian	Colorectal cancer	Digestive system cancer	1,003	1,303
Ma (31)	2020	China	Asian	Gastric cancer	Digestive system cancer	379	463
Al-Khatib et al. (32)	2020	Jordan	Caucasians	Large B-Cell lymphoma	Hematopoietic malignancy	118	228
Mao et al. (33)	2020	China	Asian	Bladder cancer	Urinary system cancer	353	433
Mhaidat et al. (34)	2021	Jordan	Caucasians	Colorectal cancer	Digestive system cancer	54	23

**Table 2:** Distribution of LEP G19A polymorphism genotype and allele.

Author	Year	Genotype distribution										HWE
		Case					Control					
		AA	AG	GG	A	G	AA	AG	GG	A	G	
Skibola et al. (16)	2004	36	169	168	241	505	119	335	351	573	1,037	0.009
Willett et al. (17)	2005	79	276	235	434	746	122	357	275	601	907	0.734
Slattery et al. (18)	2008	190	766	611	1,146	1,988	304	867	794	1,475	2,455	0.009
Doecke et al. (20)	2008	34	130	94	198	318	176	622	541	974	1,704	0.633
Tsilidis et al. (19)	2009	33	91	80	157	251	61	170	131	292	432	0.940
Wang et al. (21)	2009	39	122	92	200	306	38	119	100	195	319	0.789
Moore et al. (22)	2009	113	404	428	630	1,260	107	387	346	601	1,079	0.644
Partida-Perez et al. (23)	2010	7	44	17	58	78	25	53	24	103	101	0.691
Kim et al. (24)	2012	12	110	269	134	648	18	147	284	183	715	0.851
Zhang et al. (25)	2012	26	166	322	218	810	29	190	338	248	866	0.733
Qiu et al. (26)	2017	19	165	318	203	801	67	528	894	662	2,316	0.764
Zhang et al. (27)	2018	34	198	343	266	884	36	321	564	393	1,449	0.448
Huang et al. (28)	2018	13	71	50	97	171	29	119	111	177	341	0.089
Yang et al. (29)	2019	29	334	678	392	1,690	73	603	998	749	2,599	0.109
Lin et al. (30)	2020	51	340	589	442	1,518	59	474	767	592	2,008	0.832
Ma (31)	2020	14	120	245	148	610	30	170	263	230	696	0.883
Al-Khatib et al. (32)	2020	19	50	49	88	148	19	102	107	140	316	0.307
Mao et al. (33)	2020	11	114	228	136	570	29	162	242	220	646	0.473
Mhaidat et al. (34)	2021	10	23	21	43	65	4	11	8	19	27	0.414

HWE, Hardy–Weinberg equilibrium.

### Effect of the LEP G19A Polymorphism on Cancers

We investigated the effect of the LEP G19A mutation on cancer susceptibility in five genetic models. In all models, if the heterogeneity was less than 50%, the authors applied fixed models, whereas if the heterogeneity was greater than 50%, random models were used.

**Figure 2:**
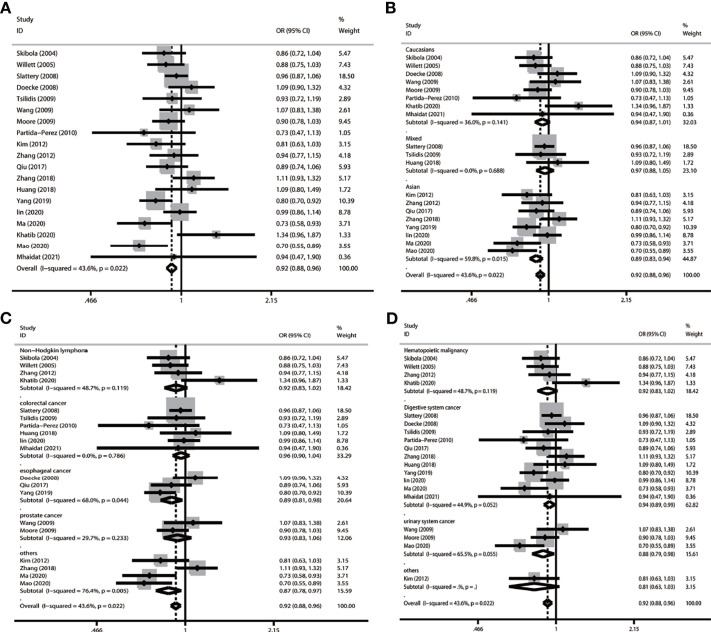
Forest plot of subgroup analysis of LEP G19A and cancer risk in the allele model (A vs. G) **(A)** LEP G19A polymorphism and overall cancer risk; **(B)** LEP G19A polymorphism and cancer risk on ethnicity; **(C)** LEP G19A polymorphism and risk of cancer type; **(D)** LEP G19A polymorphism and the risk of cancer system).

In the overall population, we found a significant relationship with cancer in four models (allele comparison: OR = 0.921, *p* = 0.000; dominant comparison: OR = 0.923, *p* = 0.004; recessive comparison: OR = 0.842, *p* = 0.000; homozygote model: OR = 0.0843, *p* = 0.001), and no relevance was observed in the heterozygote model (OR = 0.944, *p* = 0.05) (**Table 3** and **Figure 2**).

**Table 3:** The association between LEP G19A and cancer susceptibility.

G19A	No	A vs. G			AA+AG vs. GG			AA vs. AG+GG			AA vs. GG			AG vs. GG		
		*p^a^*	OR (95% CI)	*I* ^2^	*p* ^a^	OR (95% CI)	*I* ^2^	*p* ^a^	OR (95% CI)	*I* ^2^	*p* ^a^	OR(95% CI)	*I* ^2^	*p* ^a^	OR (95% CI)	*I* ^2^
Overall	19	**0.000**	**0.921 (0.883–0.961)**	43.60%	**0.004**	**0.923 (0.874–0.975)**	33.70%	**0.000**	**0.842 (0.765–0.927)**	44.10%	**0.001**	**0.843 (0.762–0.933)**	43.30%	0.050	0.944 (0.890–1.000)	29.30%
Begg’s test^b^	19	0.649			0.600			0.972			0.916			0.382		
Egger’s test^c^	19	0.963			0.802			0.460			0.587			0.907		
Ethnicity																
Caucasians	8	0.106	0.941 (0.873–1.013)	36.00%	0.337	0.951 (0.858–1.054)	0.00%	0.066	0.869 (0.749–1.009)	53.30%	0.078	0.866 (0.737–1.016)	46.70%	0.674	0.977 (0.876–1.089)	0.00%
Mixed	3	0.423	0.965 (0.885–1.053)	0.00%	0.411	1.052 (0.932–1.188)	0.00%	**0.006**	**0.785 (0.660–0.934)**	0.00%	0.056	0.833 (0.690–1.004)	0.00%	0.069	1.126 (0.991–1.280)	12.20%
Asians	8	**0.000**	0.885 (0.830–0.944)	59.80%	**0.000**	**0.862 (0.799–0.931)**	36.00%	0.119	0.866 (0.722–1.038)	54.10%	**0.039**	**0.824 (0.686–0.990)**	60.40%	**0.000**	**0.868 (0.802–0.939)**	0.00%
Cancer type																
NHL	4	0.103	0.921 (0.835–1.017)	48.70%	0.299	0.932 (0.817–1.062)	0.00%	0.082	0.832 (0.676–1.024)	69.40%	0.079	0.82 (0.658–1.023)	66.90%	0.579	0.962 (0.837–1.104)	0.00%
CRC	6	0.312	0.963 (0.896–1.036)	0.00%	0.771	1.015 (0.921–1.118)	0.00%	**0.010**	**0.816 (0.700–0.952)**	35.00%	0.081	0.863 (0.732–1.018)	0.00%	0.306	1.055 (0.952–1.168)	8.20%
EC	3	**0.014**	**0.888 (0.808–0.976)**	68.00%	**0.022**	**0.874 (0.779–0.980)**	67.30%	0.113	0.813 (0.630–1.050)	17.80%	0.100	0.801 (0.615–1.043)	52.50%	**0.060**	**0.892 (0.791–1.005)**	61.90%
PC	2	0.275	0.935 (0.828–1.055)	29.70%	0.204	0.898 (0.760–1.060)	43.90%	0.740	0.959 (0.752–1.225)	0.00%	0.485	0.911 (0.702–1.183)	0.00%	0.221	0.896 (0.752–1.068)	38.20%
Others	4	**0.010**	**0.866 (0.777–0.966)**	76.40%	**0.010**	**0.842 (0.739–0.959)**	62.50%	0.247	0.837 (0.619–1.132)	72.40%	0.132	0.791 (0.583–1.073)	76.40%	**0.018**	**0.849 (0.742–0.973)**	24.40%
System of cancer																
Hematopoietic malignancy	4	0.103	0.921 (0.835–1.017)	48.70%	0.290	0.932 (0.817–1.062)	0.00%	0.082	0.832 (0.676–1.024)	69.40%	0.079	0.82 (0.658–1.023)	66.90%	0.579	0.962 (0.837–1.104)	0.00%
Digestive system	11	**0.016**	**0.937 (0.889–0.988)**	44.90%	0.127	0.948 (0.886–1.015)	43.20%	**0.005**	**0.838 (0.740–0.949)**	44.50%	**0.028**	**0.863 (0.757–0.984)**	41.40%	0.407	0.907 (0.904–1.042)	44.70%
Urinary system	3	**0.022**	**0.881 (0.791–0.982)**	65.50%	**0.019**	**0.842 (0.729–0.973)**	50.70%	0.262	0.877 (0.698–1.103)	51.80%	0.109	0.82 (0.643–1.045)	61.20%	**0.043**	**0.855 (0.735–0.995)**	25.00%
Others	1	0.090	0.808 (0.631–1.034)	–	0.091	0.781 (0.586–1.040)	–	0.465	0.758 (0.361–1.594)	–	0.358	0.704 (0.333–1.489)	–	0.121	0.79 (0.586–1.064)	–

NO, number of study; NHL, non-Hodgkin lymphoma; CRC, colorectal cancer; EC, esophageal cancer; HWE, Hardy–Weinberg equilibrium; _, no available.

The meaning of bold values is statistically significant (P<0.05). ^a^P value of Q test for heterogeneity test; ^b^P value of Begg rank for testing publication bias; ^c^P value of Egger rank for testing publication bias.

In a subgroup analysis conducted by ethnicity, we obtained significant results in Asians in four models (allele comparison: OR = 0.885, *p* = 0.000; dominant comparison: OR = 0.862, *p* = 0.000; homozygote model: OR = 0.824, *p* = 0.039; and heterozygote comparison: OR = 0.868, *p* = 0.000); we also obtained significant results in the mixed recessive model: OR = 0.785, *p* = 0.1006. We obtained no significant results in the Caucasian population in five models (**Table 3** and **Figure 2**).

In a subgroup analysis conducted by cancer type, we obtained significant results that the LEP G19A polymorphism decreased the risk of colorectal cancer in one model (recessive model: OR = 0.816, *p* = 0.010); decreased the risk of esophageal cancer in two models (allele model: OR = 0.888, *p* = 0.014; dominant comparison: OR = 0.874, *p* = 0.022); and decreased the risk of other types of cancer in three models (allele comparison: OR = 0.866, *p* = 0.010; dominant comparison: OR = 0.842, *p* = 0.010; heterozygote comparison: OR = 0.849, *p* = 0.018) (**Table 3** and **Figure 2**).

In a subgroup analysis conducted by cancer system, we obtained significant results that the LEP G19A polymorphism decreased the risk of digestive system cancer in three models (allele comparison: OR = 0.937, *p* = 0.016; recessive comparison: OR = 0.838, *p* = 0.005; homozygote comparison: OR = 0.863, *p* = 0.028); we also obtained significant results that the LEP G19A polymorphism decreased the risk of urinary system cancer in three models (allele comparison: OR = 0.881, *p* = 0.022; dominant comparison: OR = 0.842, *p* = 0.019; heterozygote comparison: OR = 0.855, *p* = 0.043) (**Table 3** and **Figure 2**).

### Sensitivity Analysis and Publication Bias

**Figure 3:**
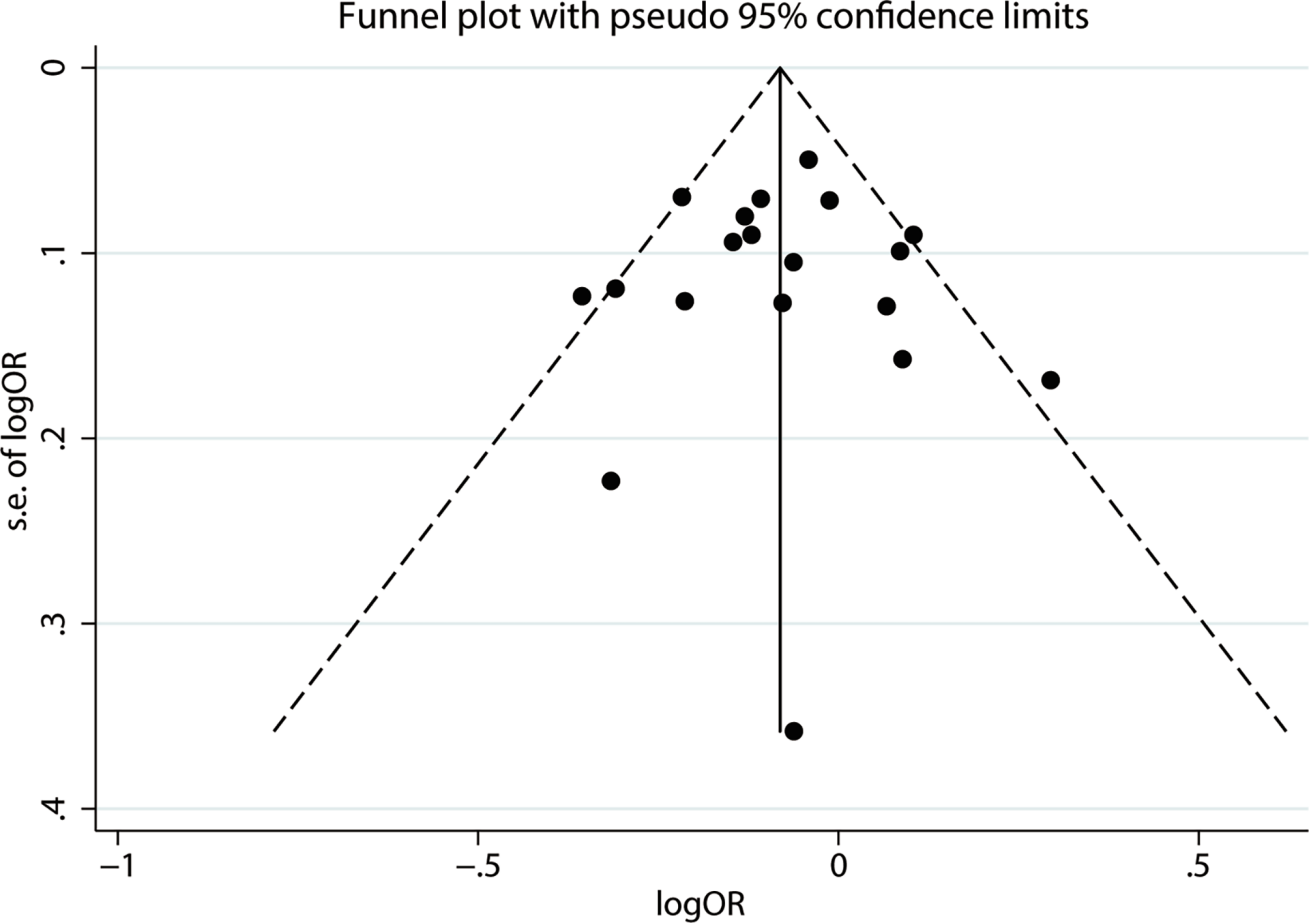
Funnel plot of publication bias on the relationship between LEP G19A polymorphism and the risk of digestion-related cancer in allele model (A vs. G).

**Figure 4:**
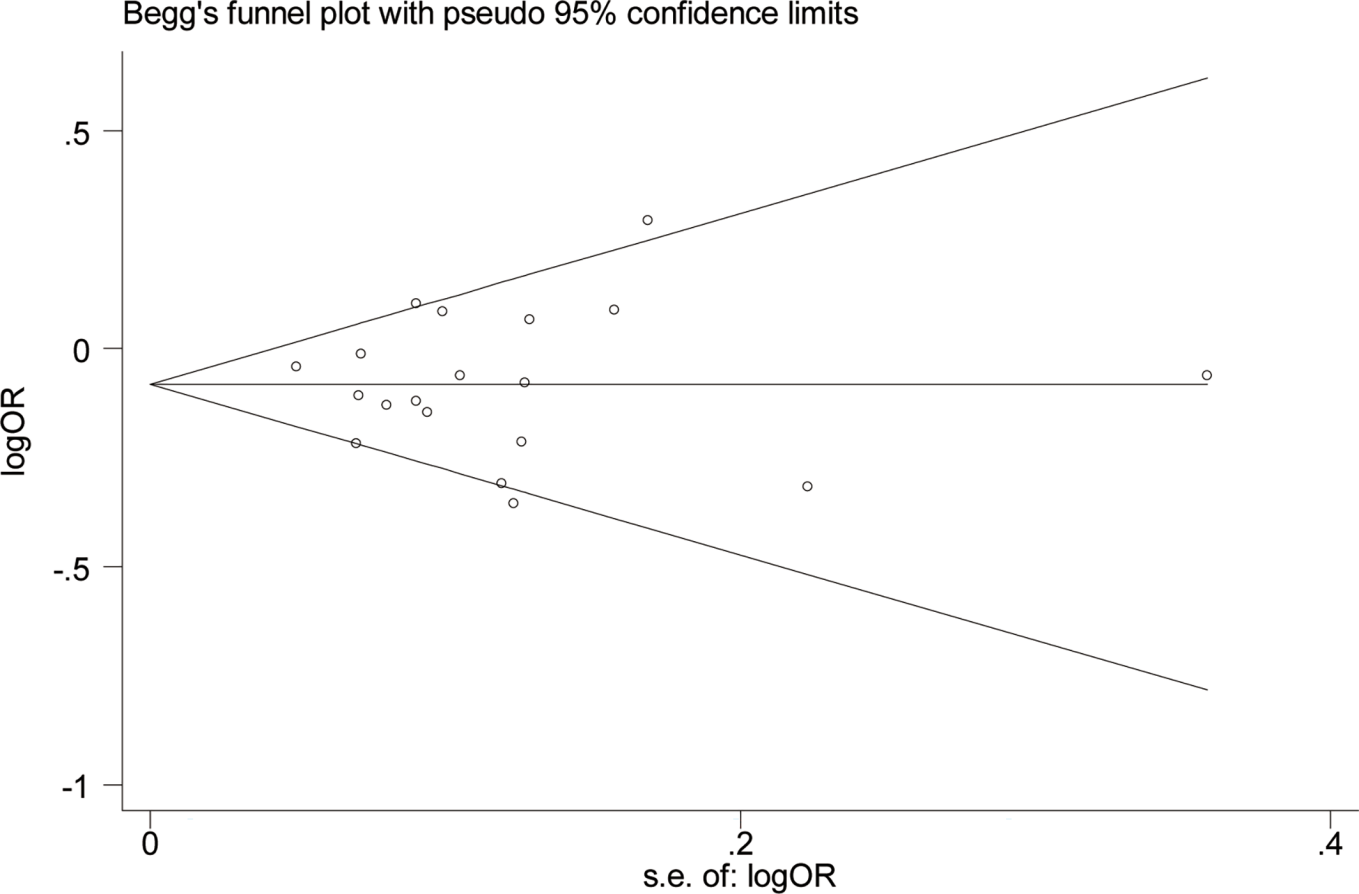
Begg’s funnel plot of meta–analysis in the allele model (A vs. G).

**Figure 5:**
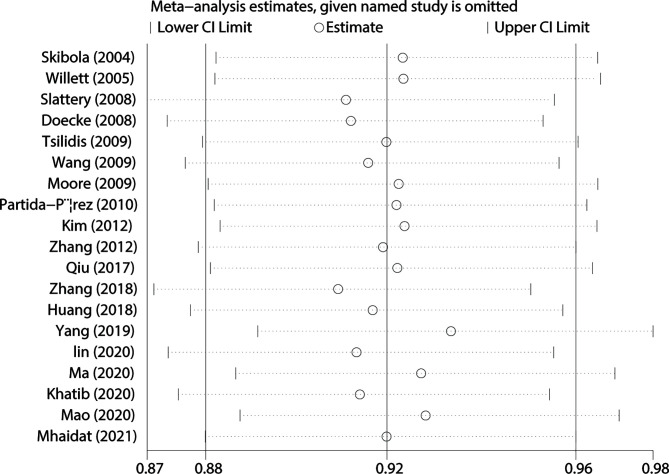
Sensitivity analysis of the influence of A vs. G comparison.

We used Begg’s and Egger’s tests to evaluate publication bias in all models. All results of Begg’s and Egger’s tests were >0.05 in all models and funnel plots, revealing that publication bias may not exist among our studies (**Table 2**, **Figures 3** and **4**). We conducted a sensitivity analysis, and pooled ORs and the corresponding 95% CIs were computed. The results did not show a significant change even though one study was deleted each time, which suggested that the results were statistically stable (**Figure 5**).

## Discussion

It has been confirmed that the occurrence of cancer is a complex, multistep, and multifactorial event that contains various genetic, environmental, and lifestyle factors, such as smoking, drinking, obesity, and genetic factor. Multiple studies have revealed that metabolic-related factors are associated with the risk of cancer (35–37). The LEP, metabolic-related factors regulating balancing by regulating acquisition and energy consumption, was confirmed relevant to cancer (38–40). The LEP G19A polymorphism may alter the transcription of mRNA and the level of LEP was confirmed to be associated with any kind of cancer (21, 22, 24–26). However, the conclusions of those studies were inconsistent. Two meta-analyses were researched by Liu et al. (41), including 10 studies, and Yang et al. (29), including 13 studies, generating conflicting results in subgroup analysis and lacking subgroup analysis of the cancer system. Meanwhile, an expanding body of literature on the relationship between LEP G19A polymorphism and cancer risk has been published. Therefore, we conducted this meta-analysis to address this relevance between the LEP G19A polymorphism and cancer risk.

Our current meta-analysis contained 19 studies of cancers containing 9,878 patients, and 14,251 controls were pooled, which contained more participants and cancer types than the previous meta-analysis. Overall, we found a significant correlation between the LEP G19A mutation and susceptibility to cancers under four models (allele model, dominant model, recessive model, and homozygote model), which means that this mutation may decrease the risk of overall cancer. This result was confirmed in a meta-analysis conducted by Liu et al. (41) and Yang et al. (29). Studies (42, 43) confirmed that the LEP G19A mutation might reduce mRNA translation with a lower serum level of LEP, which may attenuate the cancer risk as a protective factor.

Obesity was defined as an imbalance between caloric consumption and energy expenditure. Meanwhile, the LEP is a metabolic-related factor regulating balancing by regulating acquisition and consumption of energy. So it seems that obesity has a positive correlation with LEP polymorphism. However, some studies showed that there was no association between LEP polymorphism and obesity (44, 45). Mizuta et al. (46) study showed that LEP G19A was not associated with obesity. The study by Nesrine et al. (47) even showed that different polymorphisms of the LEP gene have distinct correlations with obesity. Our study showed that LEP G19A polymorphism decreases cancer risk, but the exact mechanism is unknown and mounting evidence indicates that obesity may greatly increase the risk of cancer (3–5). This provides us with a hint that LEP G19A polymorphism may not lead to cancer by gaining weight. Further studies are needed to elucidate the mechanism of action of LEP G19A polymorphism and cancer.

When stratified by ethnicity, we found a significant correlation between this mutation and Asians and no significant in Caucasians, which means that this mutation may decrease the risk of Asian people not Caucasians. This difference might be caused by a discrepancy in the interplay between genes and the environment. Moreover, the frequency of the A allele in Caucasians (68%) and Asians (44%) might be the reason for contributing to the discrepancy in the non-significant results in Caucasians. When stratified by cancer type and cancer system, it was first to describe the association between LEP G19A mutation and the cancer system. We found a significant correlation between this mutation and colorectal cancer, esophageal cancer, digestive system cancer, and urinary system cancer, which means that this mutation may decrease the risk of colorectal cancer, esophageal cancer, digestive system cancer, and urinary system cancer, but we found no correlation between this mutation and the other cancer system; the reason for this difference in risk with different tumors is as yet unknown, possibly due to LEP and its receptors playing various roles in the mediation of physiological reactions and carcinogenesis in different pathological types of cancer.

Heterogeneity may exist in our meta-analysis of cancer in the overall analysis. Stratified analyses indicated that heterogeneity was significant in some subgroups (e.g., Asians, esophageal cancer, and urinary system cancer). These factors may cause heterogeneity in our study. We checked the stability of our pooled results by sensitivity analyses. The trend of relevance was not significantly changed in the sensitivity analyses, which meant that the pooled results in our meta-analysis were statistically stable. We used Begg’s and Egger’s tests to evaluate publication bias. Begg’s and Egger’s tests’ *p*-values > 0.05 in all models, so that publication bias may exist in this meta-analysis.

The following limitations should be mentioned: (1) The number of studies focused on the relationship between LEP G19A and cancer was relatively small, so little information about stratified analyses of ethnicity, cancer type, and cancer system was available; therefore, further studies are required to determine the actual relationship in all populations. (2) Our study had no access to other potential factors influencing the results, such as other lifestyles, environments, and ages.

## Conclusion

In conclusion, this meta-analysis suggests that the LEP G19A mutation may decrease the risk of overall cancer, colorectal cancer, esophageal cancer, digestive system cancer, and urinary system cancer. In the future, more comprehensive objects containing genetic environmental interaction are warranted to discover the correlation between LEP G19A mutation and the risk of cancer.

## Data Availability Statement

The original contributions presented in the study are included in the article/supplementary material. Further inquiries can be directed to the corresponding author.

## Author Contributions

AZ, SW, FZ, WL, QL, and XL conceived the study. FZ, WL, and QL contributed to data acquisition, data interpretation, and statistical analysis. AZ, SW, and XL contributed to the study design, statistical analysis, writing, and revising of the manuscript critically. All authors contributed to the article and approved the submitted version.

## Funding

This work was supported by the National Natural Science Funds of China (82171594) and Zhao Yi-Cheng Medical Science Foundation (ZYYFY2018031).

## Conflict of Interest

The authors declare that the research was conducted in the absence of any commercial or financial relationships that could be construed as a potential conflict of interest.

## Publisher’s Note

All claims expressed in this article are solely those of the authors and do not necessarily represent those of their affiliated organizations, or those of the publisher, the editors and the reviewers. Any product that may be evaluated in this article, or claim that may be made by its manufacturer, is not guaranteed or endorsed by the publisher.
